# Transcending the Male–Female Binary in Biomedical Research: Constellations, Heterogeneity, and Mechanism When Considering Sex and Gender

**DOI:** 10.3390/ijerph19074083

**Published:** 2022-03-30

**Authors:** Stacey A. Ritz, Lorraine Greaves

**Affiliations:** 1 Department of Pathology & Molecular Medicine, Faculty of Health Sciences, McMaster University, Hamilton, ON L8S 4K1, Canada; 2 Centre of Excellence for Women’s Health, Vancouver, BC V6H 3N1, Canada; lgreaves@cw.bc.ca; 3 School of Population and Public Health, Faculty of Medicine, University of British Columbia, Vancouver, BC V6T 1Z3, Canada

**Keywords:** sex, gender, sex and gender science, biomedical research, experimental design

## Abstract

Accounting for the influences of sex- and gender-related factors on health is one of the most interesting and important challenges in contemporary health research. In biomedical research, models, experimental designs, and statistical analyses create particular challenges in attempting to incorporate the complex, dynamic, and context-dependent constructs of sex and gender. Here, we offer conceptual elaborations of the constructs of sex and gender and discuss their application in biomedical research, including a more mechanism-oriented and context-driven approach to experimental design integrating sex and gender. We highlight how practices of data visualization, statistical analysis, and rhetoric can be valuable tools in expanding the operationalization of sex and gender biomedical science and reducing reliance on a male–female binary approach.

## 1. Introduction

Understanding, accounting for, and addressing sex and gender disparities in health is arguably one of the most interesting and important challenges in contemporary biomedical research. Prior to the 1980s, most medical research ignored women and females as subjects of inquiry except when investigating ‘women’s health issues’—that is, issues directly related to reproduction, or disorders seen only or predominantly in women. In most instances, female bodies were assumed to operate in the same ways as male bodies, and findings from research conducted exclusively in men were often uncritically generalized to women. Attention to sex and, subsequently, gender as influences on biology and health gained traction in the 1980s, and by the 1990s and early 2000s many research funders, regulators, and journal editors were encouraging and mandating researchers to include considerations of sex and gender in their work [1,2,3,4,5].

Health research is a diverse enterprise, involving a wide range of methodologies, epistemologies, and disciplines. For our purposes here, we understand biomedical research to include forms of research that are experimental in nature, focused on understanding the basic cellular, molecular, and physiological mechanisms of human health and disease processes, and typically using cells, tissues, or experimental animals. The challenges of fully accounting for complex, dynamic and context-dependent constructs such as sex and gender are somewhat different in biomedical research than they are in other health research contexts. Thus, the tools and approaches used in addressing them need to be tailored and appropriate for the field. In this paper, we address these challenges from both conceptual and practical angles, with a view to improving biomedical research designs and analyses.

## 2. Sex and Gender

Until relatively recently, it was common for health researchers to use the terms ‘sex’ and ‘gender’ interchangeably. However, researchers and policy makers promoting the inclusion of sex and/or gender in health encourage people to make a distinction between them, and many have offered definitions [5,6,7,8,9,10]. In offering our own definitions here (Table 1), we synthesize key elements, and, at the same time, refine and elaborate them, drawing specific attention to their interactivity and emphasizing elements of particular importance for biomedical researchers.

At the most basic biological level, **sex** is defined by gamete size. In species where the gametes are different sizes (called anisogamy), individuals producing the smaller gamete are defined as male, and those producing the larger gamete are female [11]. Anisogamy is often (though not always) associated with variation in other structures and traits, such as chromosomal complement, endocrine function, reproductive and sexual tissues and organs, and secondary sex characteristics, as well as other physiological and morphological variations not directly related to reproductive function (such as height or body composition). We offer definitions in Table 1 but emphasize that any attempt to pin down these complex and contested terms is inherently provisional, inevitably imperfect, and open to refinement.

**Table 1:** Provisional definitions of sex and gender.

**Sex** refers to the biological attributes and functions associated with anisogamy such as chromosomal complement, endocrine function, reproductive and sexual anatomy, secondary sex characteristics, and other physiological and morphological variations and processes not directly related to reproduction (such as body size and composition, metabolic function, or organ function).	**Gender** refers to aspects of social organization that shape the range of roles, norms, behaviors, relations, aesthetics, and activities that are culturally determined to be related to presumptively belonging in a sex category. Gender encompasses the norms, roles, and institutional structures that shape any individual’s experiences, exposures, and access to power and resources, as well as one’s sense of self as a gendered individual.
Although a conceptual distinction between sex and gender is important and useful, in practice they are in dynamic dialogue with one another; in many instances, **gender/sex** can be a useful hybrid term that recognizes this entanglement [12,13].	

Although sex is typically categorized as male and female, it should be emphasized that no single attribute is necessary or sufficient to define the sex of an individual—not even gamete size. Every trait associated with sex shows some degree of variation between individuals. Some scholars have conceptualized sex as being comprised of multiple ‘layers’, only one of which is represented by the configuration of external genitalia [14]. In other words, sex consists of numerous characteristics, factors, and processes that stretch across multiple levels of biological organization, and which have normal degrees of inter- and intra-individual variation over the developmental life course. Even sex chromosome complement is not absolutely definitive. There are forms of aneuploidy that generate combinations other than XX or XY, and genetic variants can mediate reproductive development in diverse ways, as in androgen insensitivity syndrome, XX male syndrome (de la Chapelle syndrome), congenital adrenal hyperplasia, and persistent Mullerian duct syndrome, among others. Further, loss of the Y chromosome in a proportion of cells is a known occurrence with age in otherwise healthy males [15]), and many of these traits vary within the same individual across the lifespan.

Rather than thinking of sex as indicating distinct, clear-cut binary categories of male and female, it may be more accurate to consider clusters or constellations of sex-related traits instead: constellations are collections of stars that humans have grouped together as meaningful and identifiable, but no single star itself defines the constellation, nor is any single star defined by its role in the constellation; likewise, no single trait is a definitive marker of sex, and nor is any individual trait the exclusive domain of one sex. It is common to see estrogen referred to as a ‘female hormone’, in spite of the fact that estrogens are present and serve vital biological functions in all bodies (influencing fat metabolism and fluid balance, DNA repair mechanisms, cognitive function, expression of coagulation factors, and increasing muscle mass, as well as spermatogenesis and libido in males) [16]. Nor are estrogen levels clearly distinct between males and females. Prior to sexual maturation and after reproductive senescence, estrogen levels are very similar in males and females. Even in females during estrus or menstrual cycling, typical estradiol levels during certain stages overlap with the normal distribution of estradiol levels in males [17]. To use another example, even though it is often referred to as the ‘female chromosome’, the X chromosome is present and functional in every body [18]. Thus, it is misleading and not accurate to describe many traits like these as ‘female’ or ‘male’.

Moreover, reliance on male–female binary conceptualizations of sex ignores the important realities of trans individuals and those with intersex variations and differences in sexual development (DSD), who represent a non-trivial proportion of the population [19,20,21]. Understanding ‘sex’ as a constellation of various elements and holding conceptual space for biological sexes that do not conform to hegemonic norms actually helps us to better understand the influences of sex-related factors on health: by reducing our reliance on a male–female binary, we not only make our scientific work more inclusive of the various configurations of bodies, but we also enhance our understanding of the functions of *all* bodies by directing our attention to mechanism rather than category.

**Gender** can be understood as those aspects of social organization that shape the range of roles, norms, behaviors, relations, aesthetics, and activities that are deemed appropriate in relation to temporal and cultural notions of sex. At the individual level, this includes not only gender identity (that is, one’s sense of self as a gendered person), but also one’s expressions of gender through clothing, grooming, mannerisms, and speech; adherence to cultural norms of femininities and masculinities as well as appropriation of traits such as toughness, nurturance, and emotionality generally attributed to one’s gender or sex category. Gender underpins social roles in domestic, occupational, political, religious, and other spheres and the ways that one’s relationships and interactions are shaped by (gendered) norms and (gendered) policies and practices. While gender is often conceptualized as a masculine/feminine binary, there is considerable cultural and historical diversity in the ways that gender is structured, constructed, and performed.

We feel it is crucial to emphasize that gender is much more than gender identity. Activism, heightened visibility, and recognition of trans and gender diverse people over the last decade has led many people to understand that sex observed and assigned at birth is not the same as one’s gender identity. At the same time, it is important to consistently account for the many other aspects of gender described above, as these mediate many of the effects of gender on health for all people, and can be valuable for biomedical researchers to contemplate, even when not included in the experiment itself. In biomedical research, a conflation of *gender* with *gender identity* often creates confusion, leaving many researchers wondering how we would know whether lab mice or rats have an internal sense of themselves as gendered beings and how we would even begin to be able to account for that. Although we do not disregard that possibility, and there are certainly interesting questions to be asked about gender and gendered impacts and practices among animals [22], there are numerous other angles to be considered when trying to account for gender in biomedical research in addition to gender identity.

## 3. Complicating Sex and Gender

Although it is important to conceptually distinguish between sex and gender, in practice, sex and gender are in a dynamic dialogue with one another and interact in ways that highlight and generate the realities of living in sexed and gendered bodies. Such interactions can often make it difficult to draw a clear line between them. Nonetheless, it is important to include considerations of both in biomedical research. Indeed, even though gender is a social and cultural phenomenon, it is incorrect to assume that gender is not relevant for biomedical research.

Gendered experiences and ways of being in the world have material, biological impacts on the body with clear health implications. Fausto-Sterling has offered the example of bone density to illustrate this [23]. Since bones seem to be clearly biological and are affected by gonadal hormones, it can be tempting to attribute male–female differences in bone density to sex-related factors. However, there are also many gendered factors that influence bone density. Gendered occupational roles and recreational norms that can affect the type, intensity, and frequency of weight-bearing activities in which an individual participates. Similarly, exposure to sunshine stimulates vitamin D synthesis, which is important in bone homeostasis, and a wide range of gender-related factors influence sun exposure such as gendered clothing norms and religious veiling practices, health-seeking behaviors such as the use of sunscreen, and participation in outdoor occupations and recreation.

Even sex hormones themselves are affected by gender. Though our cultural narratives lead many people to believe that there is a one-way relationship where hormones drive behavior, the evidence indicates clearly that there are bidirectional influences. For example, behaving in competitive, dominant, or aggressive ways has been shown to increase testosterone [24,25,26,27], while engaging in nurturing behaviors decreases it [28,29]. In other words, the routes linking cells and society are short, and the ways that social influences permeate the body and get under the skin are myriad. Table 2 provides several examples of how gender-related influences can be translated into biology and have implications for health. Like the nature-nurture debate, it is rarely sex *or* gender determining the health outcome, as it is much more often both sex *and* gender interacting.

It is also crucial for biomedical scientists to recognize that for most sex- and gender-related factors, there is considerable overlap between the distributions for males and females. Dominant cultural narratives about gender speak about “the opposite sex”, and John Gray’s 1992 book title *Men Are From Mars, Women Are From Venus* expresses an ideology of gender essentialism with roots going back to Greek antiquity. In most of these formulations, male and female are constructed as dichotomous, opposite, innate, immutable, categorical, and binary, and treat males and females as though they are relatively homogeneous groups within themselves, ignoring the enormous degree of variability within them and the overlap between them.

Such discourses lead us to make generalizations such as “men are taller than women” or “females have lower hemoglobin than males”. Such generalizations are usually based on differences in means between populations of males and females but can obscure the fact that the actual distributions of those traits overlap considerably, may not be consistent across the lifespan, and that there is substantial heterogeneity within the categories of male and female. All of these aspects are important for biomedical researchers to consider in research design, analysis, and reporting.

The actual distribution of such data rarely supports a neat division of males and females into distinct groups. Indeed sex- and gender-related difference “commonly takes the form of *average differences* between females and males but with considerable overlap in distributions” [30]. In many instances, the sex or gender category of an individual is not a strong predictor of that individual’s behavior, expression of a trait, or response to a treatment [30,31]. While often worthy of noting, a male–female comparison showing a statistically significant difference in means does not, in itself, suggest that males and females function in fundamentally different ways, require distinct forms of treatment or intervention, or indicate that such differences are innate or natural. Rather, it is a signal that alerts us to the presence of sex- and/or gender-related factors or processes that influence the outcomes of interest, and should motivate further investigation into relevant mechanisms [32]. Ultimately, any application of that knowledge can then be directed to the mechanism itself rather than to a sex or gender category. Indeed, there are parallels between the conceptualization and operationalization of sex/gender and race, in that researchers often treat(ed) race as though it were a simple categorical variable and failed to account for the mechanisms driving racial disparities in health (principally marginalization, discrimination, and stereotyping—in other words, racism—rather than biological or genetic endowment) [33].

Implementation of interventions based on sex/gender category alone may lead to misapplication of knowledge if we overlook or fail to research the mechanisms or the distribution of the trait. For example, a Canadian study examined ferritin levels in people who were frequent blood donors and found that ferritin depletion was more common among women [34]. In response to these findings, the Canadian Blood Services implemented gender-specific recall and eligibility criteria for blood donors, lengthening the recall interval for women. However, the study did not examine any of the likely mechanisms that were likely driving the observed male–female difference in ferritin levels, such as body size, dietary intake of iron, or menstrual status. In changing the policy based on analysis of gender category alone, the new recall interval for women donors treats a 100 kg post-menopausal meat-eating woman as though she is at the same risk for ferritin depletion as a 60 kg vegetarian woman with heavy menstrual losses, and at higher risk than a 60 kg vegetarian man. The study’s data also indicate that approximately one-fifth of women who gave blood very frequently did *not* have low ferritin and could have continued to donate safely on the eight-week interval, while just over one-quarter of repeat male donors had low ferritin. Although the policy change was no doubt motivated by a sincere effort to protect the health of blood donors, the application of data from a male–female comparison without attention to mechanism or to the distribution of the data in the male and female groups results in a misapplication of the intervention: some women will see no benefit, and, simultaneously, some men who are at risk will be unprotected.

## 4. Operationalizing Sex and Gender in the Laboratory

Biomedical research incrementally builds a body of knowledge using methodologies, norms, epistemologies, and practices that lean heavily on controlled experimentation and model systems. This is a powerful configuration of tools for systematically building knowledge that has consistently delivered knowledge, discovery and advances in health. Accounting for complex, dynamic, interactive, and context-dependent constructs such as sex and gender in this context is challenging, since the power of an experiment to prove causality derives from carefully controlling variables—changing them one at a time while holding other variables constant.

Richardson has articulated an alternative way of operationalizing sex in health research that she calls *sex contextualism* [35]. Under sex contextualism, it is possible and important to recognize and “attend to variation related to sex-differentiated developmental pathways” (p17). However, she also calls on researchers to determine which sex-related factors are relevant in their particular research context, and to operationalize them appropriately for the experimental setting. For example, Richardson discusses research funded by the US Department of Defense that examined the potential use of estradiol to protect against sepsis. A sex contextualist approach to this research could extend beyond a male–female comparison by testing the effect of estradiol treatment in a sepsis model in different hormonal milieux, by looking at males, females after reproductive senescence, females in different phases of the estrus cycle, pregnant mice, ovariectomized mice, or other states with varying estrogen levels.

Under sex contextualism, Richardson notes that there is no single correct way to approach the complexity of sex in experimental research. Instead, biomedical researchers need to think carefully about which sex-related factors could plausibly influence the outcomes of interest and build their experimental design to account for *those factors themselves* as appropriate to the specific research questions being investigated.

Work with in vitro cell cultures may particularly benefit from a sex contextualist approach. A report commissioned by the US Institute of Medicine in 2001 articulated the claim that “every cell has a sex” [36], principally associated with chromosomal endowment. In saying that ‘every cell *has* a sex’, the implication is that sex is a property that exists at the level of individual cells. However, if we understand sex as an entire constellation of traits, structures, and processes across multiple levels of organization, individual cells removed from their dynamic bodily context and grown in a homogeneous population in the closed system of a flask can only embody some aspects of what we mean by sex [37].

Ritz has discussed in detail why the limitations of the in vitro environment mean that only some aspects of sex can be modelled in that context [37]. For cells living in a body, sex is not just about their chromosomal complement, but also the myriad ways their functions are tuned by inputs they receive from the cells around them, soluble mediators in the bloodstream and interstitial fluid, and modulation by the nervous system, none of which are replicated in vitro. Other standard practices in cell culture create artifacts that may interfere with the ways that sex is manifest there. For example, many culture media incorporate fetal bovine serum, which contains bovine steroid hormones [38], and the phenol red commonly present as a pH indicator is a weak estrogen mimic [39].

However, it is not pointless to try and incorporate sex considerations into cell culture research, as a sex contextualist approach can direct us in determining which specific aspects of sex can be usefully and feasibly modelled in vitro. For example, if any sex-associated hormones are thought to influence the pathways of interest, those could be added to the cultures in different physiologically-relevant doses; in doing so, it is important not to fall into the essentialist trap of equating estrogen with femaleness and testosterone with maleness, as both of these hormones are present in all bodies. It is also important to to report the sex of the donor and the chromosomal complement of the cells being used, if known, with the caveat that transformed cell lines often have unusual aneuploidies, and their chromosomal complement cannot necessarily be assumed based on the reported sex of the original donor. For example, the A549 airway epithelial cell line was derived from a male cancer patient, but the karyotype reported is hypotriploid, with one or both Y chromosomes missing in 40% of the cells that were analyzed [40]. It may be useful for researchers to review the literature to determine if there are any genes on the X or Y chromosomes that have been implicated in the pathways they are investigating and, if so, to incorporate such insights into their design, analysis, and discussion.

In experimental animal models, sex contextualism impels us to think beyond the male–female binary. Although it is important and useful to include both male and female animals in many cases, sex contextualism also asks what mechanisms could be driving male–female difference and calls for experimental designs that allow for manipulation or measurement of those causal pathways. Biomedical researchers (and health researchers more generally) should ask these kinds of questions at the hypothesis-generation stage of the research process, informed by a careful examination of the existing literature; this will allow us to build experiments that can shed light on these mechanisms, either through controlled manipulation of the pathways of interest and/or the collection of data that will shed light on mechanism.

We can usefully extend Richardson’s concept of sex contextualism to think about gender contextualism as well. Although we must be careful not to superimpose human social dynamics on to experimental animals, we may be able to model some human gender-related factors in experimental laboratory systems by manipulating variables of relevance to the biological pathways that can be affected by gender, such as those described in Table 2. For example, an allergy researcher could consider whether the human gender norms of discouraging girls from getting dirty while playing could affect allergy risk [41] by designing an experiment in which young mice are exposed to soil bacteria in their environment prior to implementing an experimental model of allergic sensitization. There are limits to this, of course, and we must be careful to resist the tendency to anthropomorphism and the naturalistic fallacy, but there may be ways we can usefully model some aspects of gender (such as activity, stimulation, risk, danger, nurturing, resources, or control) in investigating biomedical questions.

In both sex and gender contextualism, the critical shift is for researchers to take a conceptual and experimental step beyond the male–female comparison and identify a specific sex- or gender-associated *factor* to manipulate or measure that is relevant to the research context. Indeed, the epistemological strength of experimental research rests in its ability to isolate and manipulate variables in a controlled fashion, and sex and gender contextualism calls on researchers to use that strength in the service of better understanding which sex- and gender-related factors influence health, and how they operate or interact, so that we can better understand underlying mechanisms and not simply add to a descriptive catalogue of differences. There is no single strategy or approach that will be appropriate for all experimental milieux, and the operationalization of sex and gender in biomedical research will necessarily be as diverse, dynamic, and complex as are sex and gender themselves. Biomedical researchers need to grapple with developing operational definitions of sex and gender, generate hypotheses based on sex/gender mechanisms and interactions, and design experiments that isolate relevant factors and processes.

## 5. Visualization, Statistics, and Language

The ways that we discuss, visualize, and analyze data can be heavily influenced by past and present ideologies about sex and gender that introduce and perpetuate unintended stereotypes and biases in interpretation. Bringing more deliberate attention to these aspects of science can be an important contribution to the overall project of accounting for sex and gender even where it may seem difficult to address experimentally.

**Figure 1:**
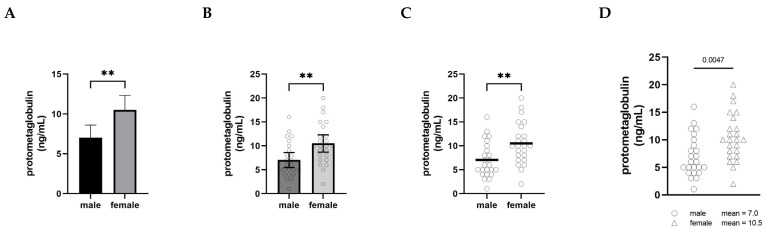
Serum levels of a fictional protein, protometaglobulin, in male and female mice. Panels (**A**–**D**) all depict the same fictional data using different visualization approaches. (**A**) is a conventional bar graph showing mean ± 95% confidence interval; (**B**) is a bar graph also showing mean ± 95% CI overlaid with a scatter plot with a dot representing each individual animal; (**C**) dispenses with the bars and confidence intervals, showing the scatter plots with a horizontal bar indicating the mean; and (**D**) shows the scatter plots only, with the means written as text beside the legend. n = 24 for each group. Groups were compared using an unpaired *t*-test; ** indicates *p* < 0.01.

The graphical presentation of data is a powerful practice for implicitly communicating narratives and interpretations. When visualizing male–female comparisons, approaches that clearly show distributions, clusters, and shapes as opposed to simply differences in means will ultimately shift interpretations and discourses as well. In Figure 1, we have plotted the same set of fictional data (serum levels of an imaginary protein, protometaglobulin) in four different ways to illustrate how different styles of visualizing a male–female comparison can affect our interpretation and appreciation of the data.

In panel A, the comparison is shown using what is probably the most common approach, a bar graph with error bars (in this case, 95% confidence intervals), and the significance of the difference between the groups is indicated with asterisks. This type of visualization creates a strong impression on the viewer of a decisive, clear difference between the levels of protometaglobulin in male and female mice.

Panel B retains the bars, confidence intervals, and asterisks, but overlays a scatter plot showing the data points for each individual. In this version, plotting each of the individual dots provides the opportunity to appreciate the range, distribution, and overlap of the individual values for each group, but the significant difference in means remains the dominant visual message.

Panel C shows the scatter plots and means only, along with the double asterisk. This makes it much easier to appreciate the extent to which the distributions overlap between the groups, and the absence of confidence intervals means that the individual data points convey the variability. At the same time, the use of the thick horizontal lines to show the means clearly conveys that the difference in means is important to pay attention to, and the asterisks show that this is a highly statistically significant difference.

Panel D depicts exactly the same data as the other panels, but this presentation of the data deemphasizes the narrative of male–female difference. By moving the group means into the legend as text, attention is entirely focused on the distribution of the individual data points—the extent of overlap between the distributions becomes the dominant visual message instead of the difference in means. The overall impression created by Panel D is notably different from the other panels. In this version, a viewer is more likely to recognize that most males and females have protometaglobulin levels in a similar range, even though the difference remains statistically significant.

None of these alternatives are intended to be prescriptive about how data *should* be visualized; rather, we include them to illustrate how choices about visualization have a profound impact on the interpretation of data in comparing males and females. That said, the visualization in Panel A is more likely to be interpreted in ways that emphasize difference, whereas Panel D more clearly highlights the overlap and similarity and is thus less likely to motivate differential treatment based only on sex or gender category.

Statistical analyses of experiments incorporating sex- and gender-related factors also need attention to ensure the selection of appropriate methods and their proper interpretation. Recently, Garcia-Sifuentes and Maney analyzed a collection of biological papers to examine their statistical treatment of sex comparisons and found that although the majority of articles analyzed claimed to have shown sex differences, appropriate statistical evidence for such claims was missing, misinterpreted, or otherwise inappropriate in 71% of them, suggesting that sex differences may be over-reported [42]. The most common way to appropriately test for a *sex x treatment* interaction is a two-way ANOVA, but these were not done in most of the papers they examined. In fact, they found that authors who did not test for interactions appropriately were over seven times more likely to claim sex-specific effects than those who did the appropriate analyses.

Part of the difficulty is that the statistical tests used in most experimental settings (such as *t*-tests and ANOVAs) are inherently geared to detecting difference between groups, not to discerning relationships or examining complex interactions between multiple factors. Given that sex and gender are complex constructs consisting of multiple factors, it may be interesting to consider whether other forms of analysis could be adapted to the experimental context, such as correlation, regression, factor analysis, or latent trait analysis, and a worthwhile area of development.

Finally, there is considerable scope for improving the language used to describe male–female comparisons, so that discussions of sex and gender in biomedical research are less prone to overgeneralization and prompt a more critical, mechanistic, precise, and incisive approach to sex- and gender-related factors in this domain. Table 3 offers examples of the ways in which language can be rearticulated. There is often a tendency to use phrases such as “sex-specific”, “sex-dependent”, or “sexual dimorphism” when describing the comparison of data from male and female groups, but in many cases these phrases may be overstating the case [42]. The words ‘specific’, ‘dimorphic’, and ‘dependent’ all evoke the idea of two distinct groups where an effect is seen in one group but not the other, but this is rarely the case for most instances where males and females are compared. “Sex difference” is also a common phrase used when comparing data from males and females. Although ‘difference’ does not evoke the idea of a clear binary with quite the same force, it is probably valuable to articulate the specific nature of the difference observed and to offer some discussion about potential mechanisms. Most often where a male–female comparison indicates some difference, it is a difference in means, and this should be specified and reflected in the wording.

The phrases “the influence of sex” or “the effect of gender” tend to suggest that ‘sex’ and ‘gender’ are simple, singular entities. In our work, we have found it useful to use the phrase “the influences of sex- or gender-related factors” because it explicitly reminds the audience that sex and gender are complex constructs composed of multiple elements. In many cases, if asked about how sex or gender might influence a given outcome, it would be common to default immediately to a male–female comparison. In contrast, if asked to think about what sex- and/or gender-related *factors* might influence a given outcome, one is explicitly directed to think about mechanisms, processes, and interactions, rather than categories. This is useful for biomedical scientists not only because it reduces reliance on male–female binaries, but also because it helps to direct attention to the relevant variables that could be incorporated into experimental designs.

## 6. Conclusions

We are on the cusp of a paradigm shift in considerations of sex and gender in biomedical health research, compelling us to transcend the use of a male–female binary comparison as the principal method of incorporating sex- and gender-related considerations. A commitment to embracing complexity and dynamism will be necessary to push sex and gender science in biomedical research forward and to avoid furthering essentialism, determinism, and categorical thinking.

Albert Einstein is often credited as saying that “everything should be made as simple as possible, *but no simpler*”. While a reliance on male–female comparisons to develop and inform our knowledge about sex, gender, and health may continue to be useful to offer initial signals and general directions, relying on it as the principal lens will hinder further progress toward the ultimate goal of gender equity in health. Sex and gender contextualism approaches can stimulate us to think beyond male–female binaries alone, and these insights can be incorporated not only in experimental design itself, but also in the language, visualization, and analyses of data. Embracing these complexities in biomedical research will facilitate navigation of the constellations of sex- and gender-related factors that impact health.

## Figures and Tables

**Table 2:** The translation of gender-related factors through biological mechanisms to health impacts.

Gender-Related Factor	Translation through Biological Mechanism	Potential Health Impact
gendered clothing styles	skin exposure to sunlight affects vitamin D synthesis, UV-induced DNA mutations shoe heel height affects stretch on Achilles tendon; narrow shoes create pressure on the hallux	effects on bone homeostasis and density immunological modulation by vitamin D skin cancer risk and location musculoskeletal pain, malformations
gendered occupational roles	exposure to chemicals, allergens exposure to infectious agents ergonomics and repetitive movements weight-bearing activity, physical demands	cancer, lung disease infection, allergy bone homeostasis, density, musculoskeletal development, injury
gendered norms of toughness	underreporting of pain, injury, delayed treatment-seeking exposure to violence leading to HPA axis activation, neurotransmitter modulation due to stress, physical injury and trauma socially-mediated modulation of testosterone production	advanced disease at diagnosis physical injury, trauma mental illness, substance use suppression of immune response changes in muscle mass
gendered norms of risk-taking	physical injury and trauma consumption and binging of substances	chronic pain, traumatic brain injury, impairment addition, liver damage, cancer risk, overdose and toxicity
gendered norms of play for children	exposure to dirt and microbes affects establishment of microbiome aerobic exercise, weight-bearing activity, physical demands neuroplasticity in response to activities demanding find and gross motor coordination, risk-taking	immune function and regulation musculoskeletal and cardiorespiratory development and function neural development, brain function

**Table 3:** Alternative formulations for describing male–female comparisons.

Problematic Formulation	Alternative Formulation
Expression of protometaglobulin appears to be sex-dependent, as serum levels were significantly higher in female mice than in males.	Most animals had levels of protometaglobulin between 5 and 15 ng/mL; 13% of female mice had levels higher than 15 ng/mL compared to 4% of males; in contrast, 25% of males had levels lower than 5 ng/mL compared to 4% of females.
A sex difference in the expression of protometaglobulin was observed, with levels approximately 50% higher in female mice than in male mice.	The mean protometaglobulin level in the group of female mice was approximately 50% higher than the mean for the male group, but there was considerable overlap between the two groups.
Sex influences the expression of protometaglobulin.	Sex-related factors appear to influence the expression of protometaglobulin.

## Data Availability

Not applicable.
